# Ivosidenib Confers BRCAness Phenotype and Synthetic Lethality to Poly (ADP-Ribose) Polymerase Inhibition in *BRCA1/2*-Proficient Cancer Cells

**DOI:** 10.3390/biomedicines13040958

**Published:** 2025-04-14

**Authors:** Danyang Zhou, Wei Liu, Yanyan Zhang, Chong Li

**Affiliations:** 1 Department of Oncology, The Affiliated Dazu’s Hospital of Chongqing Medical University, Chongqing 402360, China; danyangzhou1986@163.com (D.Z.); 152124@hospital.cqmu.edu.cn (W.L.); 150316@hospital.cqmu.edu.cn (Y.Z.); 2 Department of Respiratory, Nanjing First Hospital, China Pharmaceutical University, Nanjing 210012, China

**Keywords:** PARP inhibitors, ovarian cancer, HR repair, Ivosidenib, BRCA1/2

## Abstract

**Background/Objectives**: PARP inhibitors (PARPi) are pivotal to treating homologous recombination repair-deficient (HRD) cancers, particularly *BRCA1/2*-mutated ovarian and breast cancers. However, most ovarian and breast cancers harbor wild-type (WT) *BRCA1/2*, limiting PARPi eligibility. This study aims to identify an approved drug that could induce a BRCAness phenotype, thereby sensitizing WT BRCA cancers to PARPi. **Methods**: Ovarian and breast cancer cell lines with WT *BRCA1/2* were treated with ivosidenib. HR repair efficiency was assessed via RAD51 foci formation and reporter assays. Synthetic lethality with PARPi was evaluated using viability and colony formation assays. Mechanistic studies included RNA-binding protein pulldown, co-immunoprecipitation, and functional analyses of DNA repair pathways. YTHDC2′s role in HR was investigated through siRNA knockdown and rescue experiments. **Results**: Ivosidenib significantly reduced HR repair efficiency and sensitized cells to PARPi, inducing synthetic lethality. Mechanistically, ivosidenib directly bound YTHDC2, an m6A reader critical for HR. This interaction disrupted YTHDC2′s ability to promote DNA double-strand break repair via HR, evidenced by impaired recruitment of repair proteins (e.g., BRCA1, RAD51) and accumulation of DNA damage (γH2AX foci). YTHDC2 knockdown phenocopied ivosidenib effects, while overexpression rescued HR defects. **Conclusions**: Ivosidenib induces BRCAness in WT BRCA ovarian and breast cancers by targeting YTHDC2, thereby suppressing HR repair and enhancing PARPi sensitivity. This uncovers a novel, metabolism-independent mechanism of ivosidenib, repositioning it as a therapeutic agent for HRD tumors. These findings propose a strategy to expand PARPi eligibility to WT BRCA cancers, addressing a critical unmet need in oncology.

## 1. Introduction

Given the myriad sources of DNA damage, it is estimated that each cell in the human body experiences roughly 10,000 to 100,000 DNA lesions every day, which underpins the importance of DNA repair capability in maintaining the genome’s integrity and cell survival [1,2,3]. Poly (ADP-ribose) (PAR) polymerase (PARP) is a family of enzymes that catalyzes the addition of PAR chains to itself and other substrate proteins [4]. Among PARP family members, PARP1 is the most abundant one. Upon activation by DNA damage, PARP1 rapidly recognizes the lesions and initiates its auto-PARylation, which provides signals for the recruitment of DNA repair proteins to the site of damage, facilitating the repair of single-strand breaks (SSBs) through the base excision repair (BER) pathway [5]. Loss of function of PARP1 results in failure to repair SSBs that are subsequently converted into deleterious DNA double-strand breaks (DSBs). PARP1 inhibition-induced DNA DSBs are selectively dependent on the homologous recombination-mediated pathway for repair [6]. As a result, unrepaired DNA DSBs accumulate in cells that simultaneously lack PARP1 and HR repair, ultimately causing cell death. By taking advantage of this, the most well-known application of synthetic lethality has been developed, which involves targeting tumors with mutations in the *BRCA1* or *BRCA2* genes, which are critical for the HR pathway of DNA repair [7].

Since the approval of the first PARP inhibitor, Olaparib, for the treatment of *BRCA1/2*-mutated ovarian cancer in 2014, this class of drugs has expanded to include several others, such as rucaparib, niraparib, and talazoparib, each with varying pharmacokinetic properties and clinical applications [8,9]. These inhibitors have shown efficacy not only in ovarian cancer but also in breast, prostate, and pancreatic cancers, particularly in patients with *BRCA1/2* mutations or other defects in DNA repair pathways [10,11]. PARP inhibitors have revolutionized the approach to treating cancers with specific DNA repair deficiencies, offering a targeted and effective treatment option for patients with these challenging malignancies. Their continued development and application hold promise for improving outcomes in a broader range of cancer patients.

Despite the initial success and efficacy of PARP inhibitors, the development of resistance to PARP inhibitors poses a significant challenge, limiting their long-term therapeutic benefits [12,13,14]. Several mechanisms contribute to resistance to PARP inhibitors, allowing cancer cells to survive despite treatment. In addition, PARP inhibitors are generally less effective in cancer cells with proficient HR repair capacity.

To address these challenges, combination therapies involving PARP inhibitors have gained significant attention as a strategy to enhance therapeutic efficacy, delay resistance, and broaden the applicability of these drugs [15,16]. Here, our study aims to screen and identify FDA-approved drug that could greatly enhance PARP inhibitors’ therapeutic efficacy in *BRCA1/2*-WT cancer cells. We found that the IDH1 inhibitor ivosidenib functions as an HR repair inhibitor by targeting YTHDC2, and a combination treatment of ivosidenib and PARPi Olaparib produced synergistic anti-tumor effects in *BRCA1/2*-proficient cancer cells.

## 2. Materials and Methods

### 2.1. Cell Culture and Transfection

The *BRCA1*-WT human breast cancer cell line MDA-MB-231, the MCF-7 and *BRCA1*-WT human ovarian cancer cell line A2780, and the OVCAR-3 cell line were obtained from the American Type Culture Collection (ATCC, Gaithersburg, MD, USA) and cultured in Dulbecco’s Modified Eagle Medium (DMEM) supplemented with 10% fetal bovine serum (FBS) and 100 U/mL of penicillin–streptomycin in an atmosphere of 37 °C and 5% CO_2_. Mycoplasma contamination was checked every two weeks using Mycoplasma Detection Kit (InvivoGen, SanDiego, CA, USA). Cell transfection was performed using lipofectamine™ 3000 reagent (Thermo Fisher Scientific, Waltham, MA, USA) according to manufacturers’ instructions.

### 2.2. Colony Formation Assay

Following trypsinization and counting, cells were plated in 6-well plates at a density of 1000 cells per well. After overnight adherence, cultures were treated with either 3 μM of ivosidenib, 2 μM of olaparib, or their combination, followed by incubation for 10–14 days. Colonies were then fixed using 4% paraformaldehyde for 15 min at room temperature, washed with PBS, and stained with 0.5% crystal violet solution for 30 min. Colonies containing ≥ 50 cells were manually counted, and relative colony formation rates were calculated

### 2.3. Cell Viability Assay

Cells were plated in 96-well plates at a density of 5000–10,000 cells per well and cultured overnight to facilitate attachment. Following this incubation period, the cells were exposed to progressively increasing drug concentrations for 72 h. Cell viability was subsequently determined using the CCK-8 assay (Beyotime, Shanghai, China), with colony formation normalized relative to untreated controls.

### 2.4. Immunoflurosence Assay

First, 5 × 10^5^ cells were seeded onto glass coverslips placed in a 24-well plate and allowed to adhere overnight. After treatment with ivosidenib, Olaparib, or their combination, the cells were fixed with 4% paraformaldehyde for 10–15 min at room temperature and then permeabilized with 0.5% Triton X-100 in PBS for 10 min at room temperature, followed by washing with PBS once. After blocking with 10% goat serum for 1 h, the cells were incubated with anti-Rad51 (ABE257, 1:500 Sigma-Aldrich, Burlington, MA, USA), anti-γH2AX (05-636, 1:500, Sigma-Aldrich, Burlington, MA, USA), or anti-RPA2 (MA1-26418, 1:500 ThermoFisher Scientific, Waltham, MA, USA) diluted in blocking buffer. Then, sliders were incubated with alexa fluor^®^ 488-goat anti-mouse IgG and alexa fluor 555-goat anti-rabbit IgG antibodies (ThermoFisher Scientific, Waltham, MA, USA, 1:5000) at room temperature for 1 h in the dark. After washing three time with PBS, sliders were mounted with ProLong™ Gold Antifade mountant with DAPI(ThermoFisher Scientific, Waltham, MA, USA), and images were captured using a fluorescence microscope.

### 2.5. Immunohistochemical (IHC) Staining Assay

Tumors were collected, fixed in 10% formalin, and embedded in paraffin, followed by cutting them into 5 μm sections. Then, sliders were deparaffinized in xylene and rehydrated through a graded ethanol series (100%, 95%, 70%, 50%, and 30%), finished with a rinse in distilled water. Antigen retrieval was then performed using sodium citrate buffered by heat, and endogenous peroxidase was inactivated with 3% hydrogen peroxide in methanol for 10 min. Sliders were incubated with 10% goat FBS in PBS to block non-specific binding. Then, sliders were incubated with anti-Ki67 (Proteintech, Rosemont, IL, USA, 1:16,000) or anti-γH2AX (Sigma-Aldrich, Burlington, MA, USA, 1:200) at 4 °C overnight. After washing with PBS, the sliders were incubated with secondary antibody, and the images were taken using the R.T.U Vectastain kit (Vector Laboratories, Newark, CA, USA) according to the manufacturer’s instructions.

### 2.6. Western Blot Assay

After treatment with ivosidenib, Olaparib, or their combination, cells were harvested and lysed in RIPA buffer [50 mM Tris-HCl (pH 7.4), 150 mM NaCl, 1% NP-40, 0.5% sodium deoxycholate, 0.1% SDS] supplemented with protease and phosphatase inhibitors. Cell lysates were mixed with 5× SDS sample buffer, boiled for 5 min, and resolved through SDS-PAGE. After protein transfer onto a polyvinylidene difluoride (PVDF) membrane, membranes were blocked in 5% non-fat milk. Then, membranes were incubated with indicated primary antibodies overnight at 4 °C with rotation, followed by washing with PBST three times. Then, membranes were incubated with horseradish peroxidase (HRP)-conjugated secondary antibodies for 1 h at room temperature. After washing three times with PBST, protein expressions were detected using an enhanced chemiluminescence (ECL) substrate.

### 2.7. Comet Assay

The comet assay (single-cell gel electrophoresis) was performed using the OxiSelect™ Comet Detection Kit (ENZO Life Science, Farmingdale, NY, USA, #ADI-900-166) according to the manufacturer’s instructions. Briefly, after treatment, cells were suspended in PBS and mixed with 100 μL of OxiSelect™ comet agarose and immediately transferred to OxiSelect™ comet slides. Slides were immersed in cold lysis buffer (2.5 M NaCl, 100 mM EDTA, 10 mM Tris, 1% Triton X-100, pH 10) at 4 °C for 2 h. Slides were then incubated in freshly prepared alkaline buffer (300 mM NaOH, 1 mM EDTA, pH > 13) for 20 min at 4 °C to allow DNA unwinding, followed by electrophoresis in TBE buffer at 30 V for 10 min. After electrophoresis, the slides were fixed in 70% ethanol for 5 min and stained with 1 × Vista Green DNA dye for 30 min at room temperature in the dark after drying. Then, images were collected using a fluorescence microscope (Olympus, Tokyo, Japan), and tail DNA percentage was analyzed using OpenComet v1.3.1 software.

### 2.8. Xenograft Tumor Study

Six-week-old female nude mice were purchased from GemPharmatech Co., Ltd. (Nanjing, China) and maintained under specific pathogen-free conditions. All animal experiments were conducted in accordance with institutional ethical guidelines and approved by the Institutional Animal Care and Use Committee (IACUC) of Dazu Hospital of Chongqing Medical University. Then, 100 mg/kg of ivosidenib, 100 mg/kg of Olaparib, or their combination were orally administrated to mice when tumor volumes reached around 100 mm^3^. Tumor volume was monitored every three days and calculated through the equation V = (L × W^2^)/2 (V, volume; L, length; W, width).

### 2.9. Homologous Recombination (HR) Repair Assay

U2OS-DRGFP cells containing a single copy of DR-GFP reporter construct that contains a GFP gene disrupted by an I-SceI recognition site were used to measure HR repair efficacy, as previously described. Briefly, U2OS-DRGFP cells were transfected with pCBA-SceI using lipofectamine 3000 (Thermo Fisher Scientific). Then, 24 h after transfection, cells were treated with increasing concentrations of ivosidenib. Another 24 h later, GFP-positive cells, indicative of HR repair events, were analyzed through flow cytometry.

### 2.10. Drug Affinity Responsive Target Stability (DARTS)

First, 500 μg of A2780 cell lysates was incubated with or without indicated concentrations of ivosidenib at room temperature for 1 h. Then, cell lysates were treated with or without pronase (5 μg/mL) at room temperature for 20 min. After adding SDS-PAGE loading buffer, samples were resolved through SDS-PAGE, and the stability of YTHDC2 was analyzed through Western blotting.

### 2.11. Statistics

GraphPad Prism 7 software was used for statistical analysis. The statistical significance of differences between groups was analyzed using the two-sided unpaired Student’s *t*-test. *p* < 0.05 indicates that the differences are statistically significant.

## 3. Results

### 3.1. High-Throughput Screening Identifies Ivosidenib as a PARPi Sensitizer in BRCA1/2 Wild-Type Ovarian Cancer Cells

**Figure 1:**
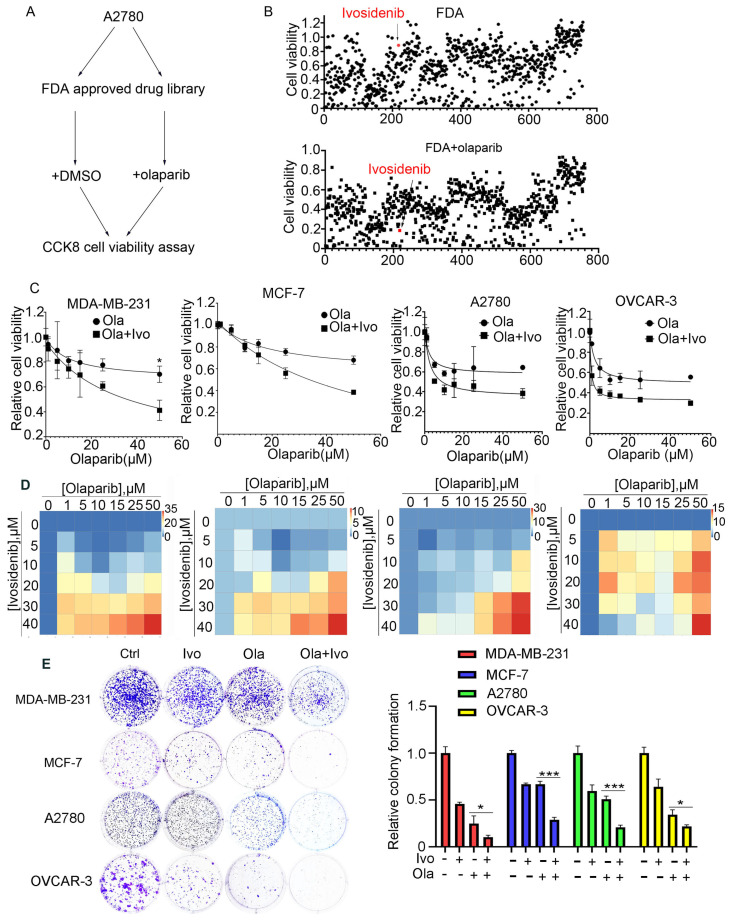
High-throughput screening shows that IDH1 inhibitor ivosidenib greatly sensitizes BRCA1/2-proficient cancer cells to PARPi. (**A**) The workflow for screening sensitizer drugs for the PARP inhibitor Olaparib in A2780 cells using an FDA-approved drug library (TargetMol, #L1000) that contains 2040 approved small molecule drugs. (**B**) The relative cell viability of A2780 cells grown in the presence of 10 μM of FDA-approved drugs alone (upper) or FDA-approved drugs plus 10 μM of Olaparib for 72 h; cell viability was analyzed through CCK-8 assay. (**C**) Ivosidenib increases Olaparib sensitivity in *BRCA1/2*-WT breast and ovarian cancer cells. Indicated cells were treated with increasing concentrations of Olaparib (ola) in the presence or absence of 5 μM of ivosidenib (Ivo) for 72 h, followed by cell viability analysis. (**D**) Ivosidenib and Olaparib synergistically suppress cancer cell growth. Indicated cells were co-treated with serial dosages of Olaparib and ivosidenib for 72 h, followed by Bliss synergy analysis. (**E**) Ivosidenib and Olaparib synergistically inhibit clonogenic survival in *BRCA1/2*-WT cancer cells. The colony formation analysis indicated cells treated with 2 μM of ivosidenib, 2 μM of Olaparib, or their combination. Data are presented as means ± SD, *n* = 6. * *p* < 0.05 and *** *p* < 0.001 based on the two-tailed *t*-test.

*BRCA1/2* gene mutations predict PARPi sensitivity; however, the majority of cancer patients carrying normal *BRCA1/2* genes are not eligible for PARP inhibitors. To extend the application of PARPi to *BRCA1/2* wild-type (WT) cells and develop a feasible PARPi combination therapy strategy, we employed an FDA-approved drug library and treated A2780 cells bearing normal *BRCA1/2* with FDA-approved drugs alone or with a low dose of PARPi Olaparib for 73 h, followed by an analysis of cell viability using CCK-8 (Figure 1A). Through screening, we found that ivosidenib greatly potentiated Olaparib-induced inhibition of cell viability (Figure 1B). Ivosidenib is a mutant isocitrate dehydrogenase 1 (IDH1) inhibitor that is clinically used to treat patients with relapsed or refractory acute myeloid leukemia (AML) with IDH1 mutation [17,18].

To further confirm the ivosidenib-mediated sensitization of cancer cells to PARPi, the *BRCA1/2*-WT breast cancer cell line MDA-MB-231, MCF-7, and *BRCA1/2* WT ovarian cancer cell A2780, OVCAR-3 cells were treated with increasing concentrations of Olaparib in the presence or absence of 2 μM of ivosidenib. As shown in Figure 1C, ivosidenib significantly enhanced Olaparib-induced cell growth inhibition in all of the above cells. Next, the synergism between Olaparib and ivosidenib in the above cells was analyzed, and, as expected, Olaparib and ivosidenib synergistically suppressed cancer cell growth (Figure 1D). Their synergistic anti-tumor efficacy was further confirmed through clonogenic survival analysis (Figure 1E). Thus, these results indicate that mutant IDH1 inhibitor ivosidenib remarkably potentiates therapeutic efficacy of PARPi either in *BRCA1*-WT or mutant cancer cells.

### 3.2. Ivosidenib Enhances Olaparib-Induced DNA Double-Strand Breaks (DSBs) in Cancer Cells

**Figure 2:**
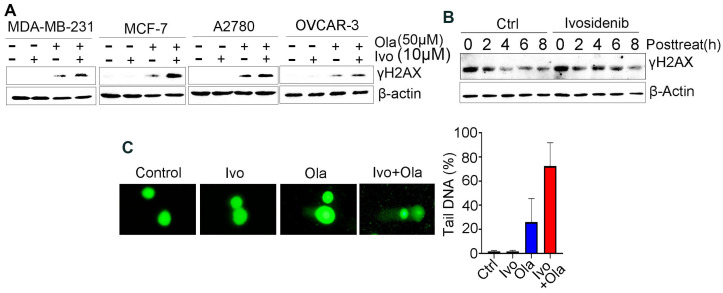
Ivosidenib (Ivo) greatly potentiates Olaparib (Ola)-induced DNA damage in *BRCA1/2*-proficient cells. (**A**) Ivosidenib enhances Olaparib-induced γH2AX formation in *BRCA1/2*-WT cancer cells. Indicated cells were treated with 50 μM of Ola, 10 μM of Ivo, or their combination for 48 h, followed by Western blot analysis of γH2AX. (**B**) Ivosidenib delayed the reduction of γH2AX after Olaparib treatment. A2780 cells were treated with 100 μM for 48 h and then released into normal culture medium in the presence or absence of Ivo for indicated time points, followed by Western blot analysis of γH2AX. (**C**) Ivosidenib increases Olaparib-caused DNA damage. A2780 cells were treated with 50 μM of Ola, 10 μM of Ivo, or their combination for 48 h, followed by measuring the DNA damage through comet assay. Data are presented as means ± SD.

Given that PARPi kills cancer cells through the induction of deleterious DNA DSBs [19], we examined whether an ivosidenib/Olaparib combination could induce a greater level of DSBs. We examined γH2AX, which is the phosphorylation of Ser139 of histone H2AX and well-recognized as the biomarker of DSB [20], in *BRCA1*-proficient cancer cells. As expected, ivosidenib alone did not induce obvious DNA damage in breast or ovarian cancer cells; however, ivosidenib greatly increased Olaparib-induced γH2AX in cancer cells (Figure 2A). Meanwhile, ivosidenib treatment did not obviously affect phosphorylation of Chk1 and Chk2 upon ion irradiation (IR) (Figure S1), indicating that ivosidenib could not inhibit DNA damage response signaling.

To examine whether the increase in DNA DSBs resulted from impaired repair capacity, we next tested γH2AX’s disappearance at different time points after Olaparib treatment in the presence or absence of ivosidenib. Interestingly, we found that ivosidenib treatment significantly delayed the clearance of Olaparib-induced γH2AX (Figure 2B), which indicates that the increased γH2AX in ivosidenib/Olaparib co-treated cells resulted from impaired repair capacity. Additionally, consistent with γH2AX analysis, the comet assay also confirmed that the combination treatment of ivosidenib and Olaparib significantly increased DNA damage compared with Olaparib alone (Figure 2C). These results demonstrate that ivosidenib treatment impairs the repair of PARPi-induced DSBs.

### 3.3. Ivosidenib Inhibits Homologous-Recombination (HR) Repair

**Figure 3:**
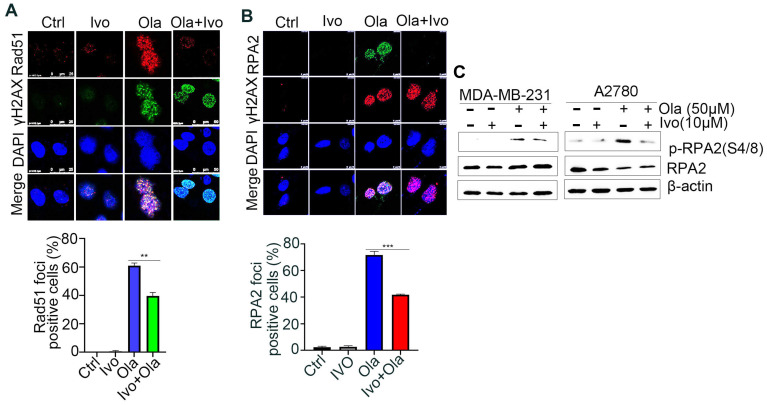
Ivosidenib (Ivo) treatment suppresses the repair of Olaparib (Ola)-induced DSBs. Ivosidenib inhibits HR repair. (**A**,**B**) Ivosidenib A2780 cells were treated with 50 of μM Ola, 10 μM of Ivo, or their combination for 48 h, followed by immunofluorescence analysis of Rad51/γH2AX (**A**) or RPA2/γH2AX (**B**). (**C**) Cells were treated as indicated for 48 h, followed by Western blot analysis of pRPA2 S4/8. Data were are presented as means ± SD. ** *p* < 0.01 and *** *p* < 0.001 according to the two2-tailed *t*-test.

PARPi-induced DSBs selectively rely on the HR-mediated pathway for repair [21]. Rad51 recombinase-guided strand invasion is essential to complete the HR repair process, and Rad51 accumulation at DSB sites is considered one of the biomarkers of HR repair [22]. As shown in Figure 3A and Figure S2A, ivosidenib treatment significantly inhibited Olaparib-induced Rad51 foci in *BRCA1/2*-proficient A2780 and MDA-MB-231 cells, indicating that ivosidenib could suppress HR repair. DNA end-resection that creates single-stranded DNA overhang is the rate-limiting step of HR repair [23]. Once resection is initiated, the replication protein A (RPA) complex is quickly coated on the resected single-strand DNA, and RPA2 foci formation is considered the biomarker of DNA end-resection [24]. Consistent with the Rad51 foci, a significantly lower level of RPA2 foci was observed in A2780 cells treated with the ivosidenib/Olaparib combination compared with cells treated with Olaparib alone (Figure 3B). Once resected DNA is coated by the RPA complex, RPA2 is then phosphorylated by ATR at its Ser4/8; thus, RPA2 phosphorylation at S4/8 is also known as the DNA end-resection marker [25]. Similarly, ivosidenib treatment also remarkably inhibited Olaparib-induced RPA2 S4/8 phosphorylation (Figure 3C). Thus, these results demonstrate that ivosidenib inhibits HR repair and may confer a BRCAness phenotype to *BRCA1/2*-proficient cancer cells.

### 3.4. Ivosidenib Targets YTHDC2

**Figure 4:**
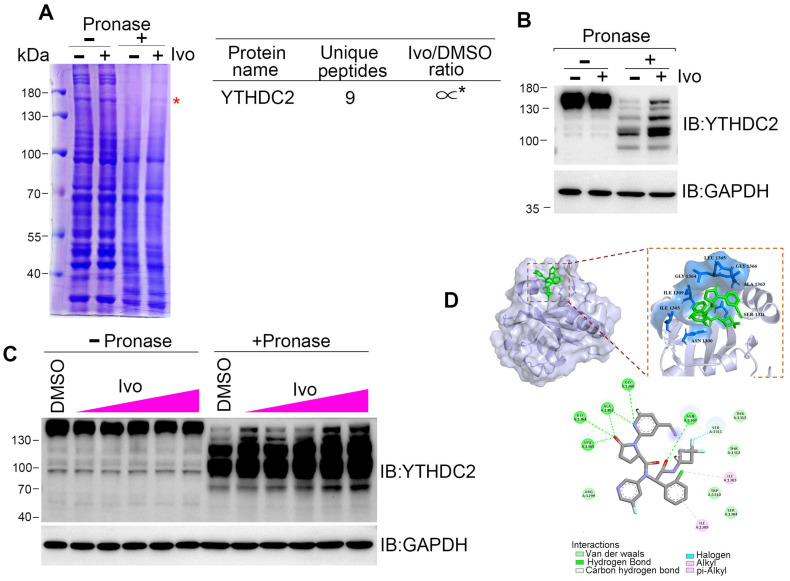
Ivosidenib targets YTHDC2. (**A**–**C**) Ivosidenib protects pronase-mediated digestion of YTHDC2. (**A**) 200 μg of A2780 cell lysates was incubated with/without 10 μM of ivosidenib (Ivo) before being subjected to pronase digestion; the protein contents were analyzed through Coomassie staining and mass spectrometry analysis. * indicates the protein YTHDC2. The drug affinity responsive target stability (DARTS) detection of single-dose (**B**) or serial-dosage (**C**) Ivo prevention of pronase-mediated YTHDC2 digestion. (**D**) Molecular docking shows the binding pocket of Ivo in YTHDC2 protein.

HR repair is executed by several repair machinery proteins, including BRCA1, CtIP, Mre11, Rad51, and RPA2 [26]; however, ivosidenib treatment did not affect the expression of these proteins (Figure S2B). To explore how ivosidenib suppresses HR repair, we employed the drug affinity responsive target stability (DARTS) technique for target identification to uncover its intracellular target [27]. Coomassie blue staining showed that a protein between 130 and 180 kDa was protected from pronase-mediated proteolysis after ivosidenib treatment, and the subsequent mass spectrometry (MS) analysis revealed that RNA m6A reader protein YTHDC2 was enriched in ivosidenib-treated cell lysates (Figure 4A). We then utilized Western blotting to confirm ivosidenib-mediated proteolytic protection of YTHDC2. As shown in Figure 4B, ivosidenib treatment significantly protected YTHDC2 proteolysis. Moreover, ivosidenib-concentration-dependent proteolytic protection of YTHDC2 was also observed (Figure 4C), which indicates that ivosidenib interacts with YTHDC2. Molecular docking analysis showed that ivosidenib is predicted to associate with the C-terminal of YTHDC2, which functions as the RNA-binding domain [28].

### 3.5. YTHDC2 Is Required for Proficient HR Repair

**Figure 5:**
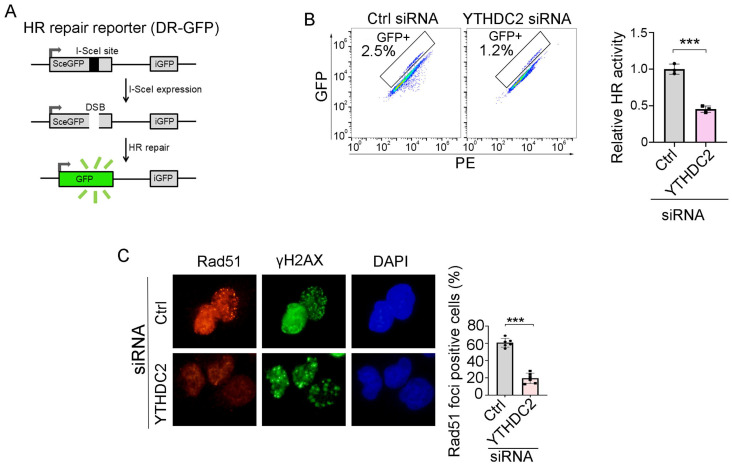
YTHDC2 knockdown suppresses HR repair. (**A**) Schematic diagram of HR repair reporter system (DR-GFP). (**B**,**C**) Silencing of YTHDC2 decreases HR repair. QU20S-DRGFP cells were transfected with control (ctrl) or YTHDC2 siRNA and, 24 h later, cells were transfected with pCBASceI plasmid to induce endonuclease-induced DSB, followed by flow cytometry analysis of GFP expression 48 h after pCBASceI expression (**B**). A2780 cells were transfected with control (Ctrl) or YTHDC2 siRNA; cells were then treated with 50 μM of Olaparib for 48 h, followed by immunofluorescence analysis of Rad51/γH2AX (**C**). Data are presented as means ± SD. *** *p* < 0.001 according to the two-tailed *t*-test.

We next sought to determine whether ivosidenib suppresses HR repair by targeting YTHDC2. We utilized the HR repair reporter system DR-GFP, in which the GFP gene is interrupted by the I-SceI endonuclease cutting site and the functional GFP can only be restored through HR repair using the downstream iGFP sequence as the template after I-SceI expression [25], to measure whether YTHDC2 participates in HR repair (Figure 5A). As shown in Figure 5B, knockdown of YTHDC2 significantly inhibited HR repair. Consistent with the reporter assay, YTHDC2 knockdown also caused a reduction of Rad51 foci in *BRCA1*-proficient A2780 cells (Figure 5C). Thus, these results demonstrate that YTHDC2 promotes HR repair.

### 3.6. Ivosidenib Inhibits HR Repair by Targeting YTHDC2

**Figure 6:**
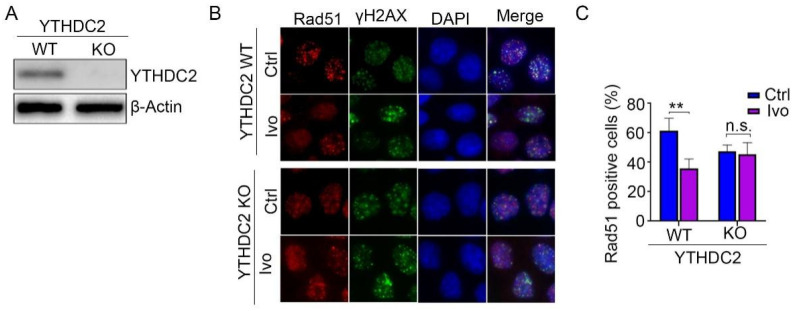
Depletion of YTHDC2 abolished ivosidenib-induced reduction of Rad51 foci formation. (**A**) YTHDC2 was depleted in A2780 cells using the CRISPR/Cas9 technique. (**B**,**C**) Ivosidenib inhibits Rad51 foci by targeting YTHDC2. WT or YTHDC2 knockout A2780 cells were pre-treated with or without 10 μM of ivosidenib (Ivo) for 2 h; then, cells were irradiated with 2 Gy X-ray, and Rad51/γH2AX foci formation was then analyzed. The representative Rad51/γH2AX staining (**B**) and quantification of Rad51 positive cells (**C**) were shown. Data are presented as means ± SD. ** *p* < 0.01 according to the two-tailed *t*-test. n.s., no significance.

To test whether the HR repair inhibiting activity of ivosidenib occurs by targeting YTHDC2, we depleted YTHDC2 protein expression in A2780 and MCF7 cells (Figure 6A and Figure S3A). Then, WT and YTHDC2-depleted cells were treated with Olaparib in the presence or absence of ivosidenib. The following Rad51 immunofluorescence staining assay showed that ivosidenib significantly inhibited Rad51 foci formation in WT cells but not in YTHDC2-depleted cells (Figure 6B,C and Figure S3B,C), which demonstrates that ivosidenib targets YTHDC2 to inhibit HR repair.

### 3.7. Ivosidenib Sensitizes BRCA1/2-Proficient Cancer Cells to PARPi In Vivo

**Figure 7:**
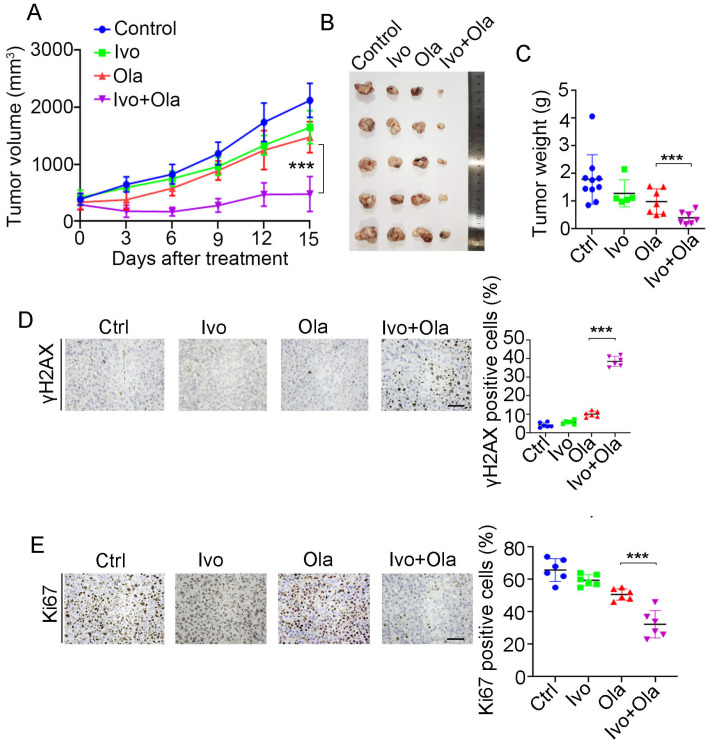
Combination treatment of ivosidenib (Ivo) and Olaparib (Ola) produces synergistic anti-tumor efficacy in *BRCA1/2*-proficient ovarian cancers. (**A**–**C**) Ivosidenib and Olaparib synergistically suppress tumor growth. A2780 xenograft bearing mice treated with Ola (100 mg/kg, orally) and Ivo (100 mg/kg, orally) and tumor growth curve (**A**), tumor image (**B**), and tumor weights (**C**) are shown. (**D**,**E**) Co-treatment with ivosidenib and Olaparib greatly induces DSBs and reduces proliferation in A2780 xenograft tumors. Immunohistochemistry analysis of γH2AX (**D**) and Ki67 (**E**) in tumors treated as indicated. Data are presented as means ± SD. *** *p* < 0.001 according to the two-tailed *t*-test.

Given that HR repair activity dictates PAPRi’s therapeutic efficacy and ivosidenib suppresses HR repair via YTHDC2, we evaluated the therapeutic efficacy of Olaparib alone, ivosidenib alone, and their combination in the A2780 xenograft. As shown in Figure 7A, the combination of ivosidenib and Olaparib strikingly inhibited the growth of A2780 xenograft tumors, while either ivosidenib or Olaparib alone mildly retarded A2780 tumor growth (Figure 7A–C). Consistent with the in vitro clonogenic survival assay, there was a remarkable increase of γH2AX in ivosidenib/Olaparib co-treated tumors compared with tumors treated with ivosidenib or Olaparib alone (Figure 7D). Consequently, a lower level of Ki67 was observed in tumors that received the ivosidenib/Olaparib combination (Figure 7E).

## 4. Discussion

As the first FDA-approved anti-cancer drug that exploits the concept of synthetic lethality, PARP inhibitors (PARPi) are widely employed to treat breast, ovarian, pancreatic, and prostate cancer patients associated with *BRCA1* and *BRCA2* mutations [8]. Despite PARPi obtaining great success and significantly improving patients’ survival, its resistance has emerged and limited its long-term efficacy [13]. Several mechanisms, including the restoration of HR repair capability, the reverse mutation of *BRCA1/2,* or the loss function of 53BP1, have been identified that lead to de novo or acquired PARPi resistance [29,30]. In addition, PARPi is not eligible to treat cancer patients carrying normal *BRCA1/2*. Therefore, combination strategies are urgently needed to extend its application and counteract resistance. To design a rational PARPi combination therapy, we screened a library of FDA-approved drugs and identified the mutant IDH1 inhibitor ivosidenib, which greatly potentiates PARPi efficacy in either *BRCA1/2* wild-type (WT) or mutant cancer cells. A combination treatment of ivosidenib and PARPi Olaparib produced synergistic therapeutic effects in vitro and in a xenograft ovarian cancer model. Ivosidenib targets the mutant metabolic enzyme IDH1 and suppresses cancer growth by normalizing the metabolic pathway. Whether ivosidenib possesses anti-cancer functions beyond metabolic regulation remains unexplored. Here, we found that ivosidenib negatively regulates HR-mediated DSB repair by targeting YTHDC2. Our results are in agreement with recent a report that shows that ivosidenib synergized with conventional chemotherapeutics in pancreatic cancer [31]. Despite the fact that ivosidenib is an FDA-approved anti-cancer drug that was used to treat acute myeloid leukemia (AML) and cholangiocarcinoma [18], the therapeutic efficacy of the combination of ivosidenib and PARPi needs to be further investigated in clinical trials. Thus, our study provides a novel and rational combination strategy to optimize PARPi therapies.

Ivosidenib is an oral anti-cancer agent that is well-recognized for its ability to target the mutant IDH1 enzyme to reduce the production of the oncometabolite 2-hydroxyglutarate (2-HG), consequently alleviating its oncogenic effects, including epigenetic alteration and metabolism reprograming [32,33]. Although ivosidenib is selectively used to cure cancer patients carrying IDH1 mutations, it still exerts a suppressive function on cancer cells with normal IDH1 [34], which implies that ivosidenib has a secondary intracellular target beyond mutant-*IDH1*-mediated metabolic regulation. However, direct known targets beyond mutant *IDH1* have not been identified yet. Here, we employed drug affinity responsive target stability (DARTs) technique and identified that ivosidenib directly associates with N6-Methyladenosine reader YTHDC2. And, we further showed that ivosidenib inhibits HR repair by targeting YTHDC2. Ivosidenib treatment confers an HR repair deficient phenotype and PARPi hypersensitivity to BRCA1/2-WT cancer cells. Therefore, our present study uncovered a novel biological function of ivosidenib, which might extend its future clinical application.

HR repair deficiency predicts PARPi sensitivity, and PARPi specifically induces DNA double-strand breaks (DSBs) in HR repair deficient cells. Although numerous small molecules have been reported as HR repair inhibitors [35], our study aimed to identify an HR repair suppressive compound from an FDA-approved drug library and successfully identified that ivosidenib potently inhibits HR repair in ovarian and breast cancer cells. Ivosidenib greatly potentiated Olaparib-induced DSBs in *BRCA1/2*-WT or mutant cancer cells. Taken together, our study employed high-throughput screening using an FDA-approved drug library to identify a potential PARPi sensitizer and discovered that the mutant *IDH1* inhibitor ivosidenib greatly potentiates the therapeutic effects of PARPi. Importantly, we discovered that ivosidenib inhibits HR repair activity by directly targeting YTHDC2, which provides a novel combination strategy to optimize PARPi therapy in the future.

## 5. Conclusions

Through comprehensive screening, we demonstrated that the mutant IDH1 inhibitor ivosidenib significantly enhances the sensitivity of BRCA1/2-proficient breast and ovarian cancer cells to PARP inhibitors (PARPi). Subsequent mechanistic investigations revealed that ivosidenib directly targets the m6A reader protein YTHDC2 in IDH1 wild-type cancer cells, thereby impairing homologous recombination (HR) repair capacity in these BRCA-competent malignancies. Importantly, our findings suggest that this FDA-approved agent may induce a BRCAness phenotype through YTHDC2-mediated HR suppression. These results provide a preclinical rationale for combining ivosidenib with PARPi to overcome therapeutic resistance in BRCA1/2-proficient cancers. Future directions should prioritize (1) the validation of the combination’s efficacy using patient-derived xenograft (PDX) models; (2) systematic assessment of safety and pharmacokinetic profiles in preclinical models; and (3) elucidation of the molecular cascade linking YTHDC2 inhibition to HR repair dysfunction, particularly its interplay with DNA damage response pathways.

## Data Availability

The data presented in this study are available from the corresponding author on reasonable request.

## Supplementary Materials

The following supporting information can be downloaded at: https://www.mdpi.com/article/10.3390/biomedicines13040958/s1, Figure S1: Ivosidenib(Ivo) treatment did not affect DNA damage response signaling; Figure S2: Ivosidenib(Ivo) suppresses HR repair; Figure S3: Depletion of YTHDC2 diminishes Ivosidenib-mediated suppression of HR repair.
