# Supplementary material for: Ivosidenib Confers BRCAness Phenotype and Synthetic Lethality to Poly (ADP-Ribose) Polymerase Inhibition in BRCA1/2-Proficient Cancer Cells
Source: Biomedicines. 2025 Apr 14;13(4):958. doi: 10.3390/biomedicines13040958 (PMC12025137; doi:10.3390/biomedicines13040958)
Supplement: Supplementary file 1 [file biomedicines-13-00958-s001.zip › biomedicines-3524239-supplementary.pdf]

Supplementary Figures

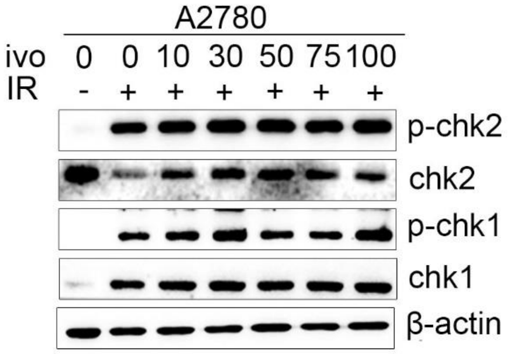

Figure S1. Ivosidenib(Ivo) treatment did not affect DNA damage response signaling. A2780 cells were pre-treated with increasing concentrations of Ivo for 2 hours, followed by IR treatment (2 Gy), then western blot analysis of Chk1 and Chk2 phosphorylation at 2 hours after radiation.

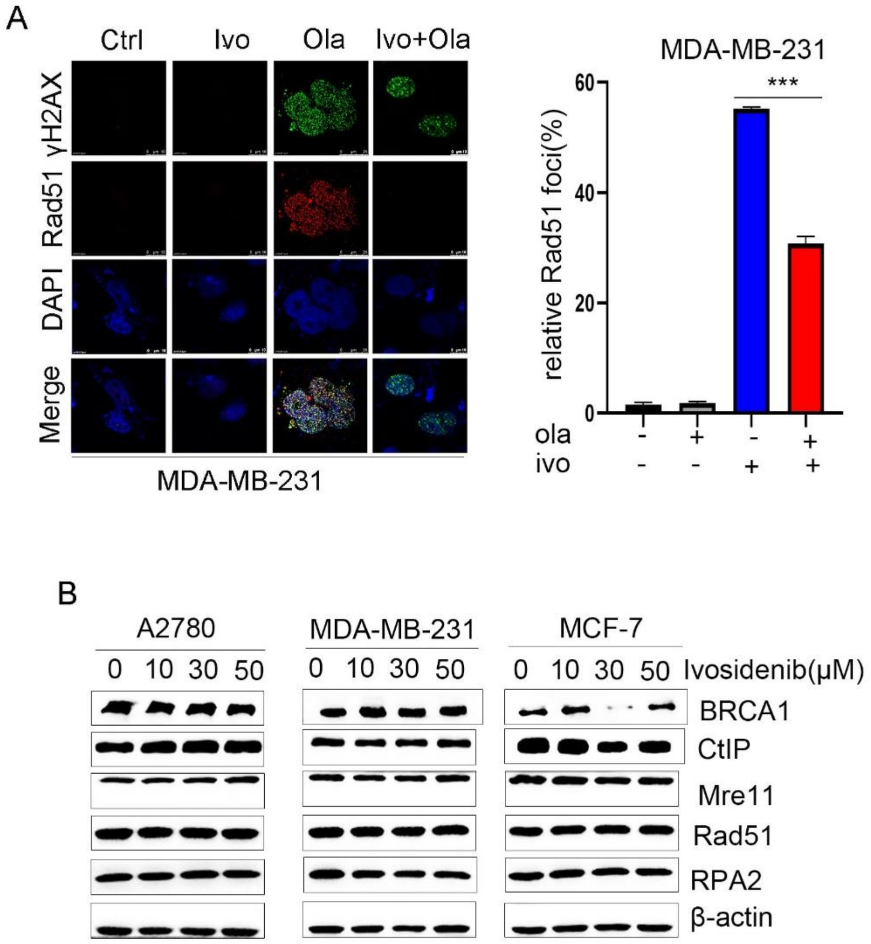

Figure S2. Ivosidenib(Ivo) suppresses HR repair. (A) Immunofluorescence analysis of Rad51/γH2AX foci in MDA-MB-231 cells treated with Ivosidenib(Ivo, 10 μM), Olaparib (Ola, 50μM) or their combination. **(B)** BRCA1/2 proficient cells were treated with indicated concentrations of Ivo for 24 hours, followed by western blot analysis of indicated protein levels. Data were presented as means ± SD. ***p<0.001 by 2-tailed t-test.

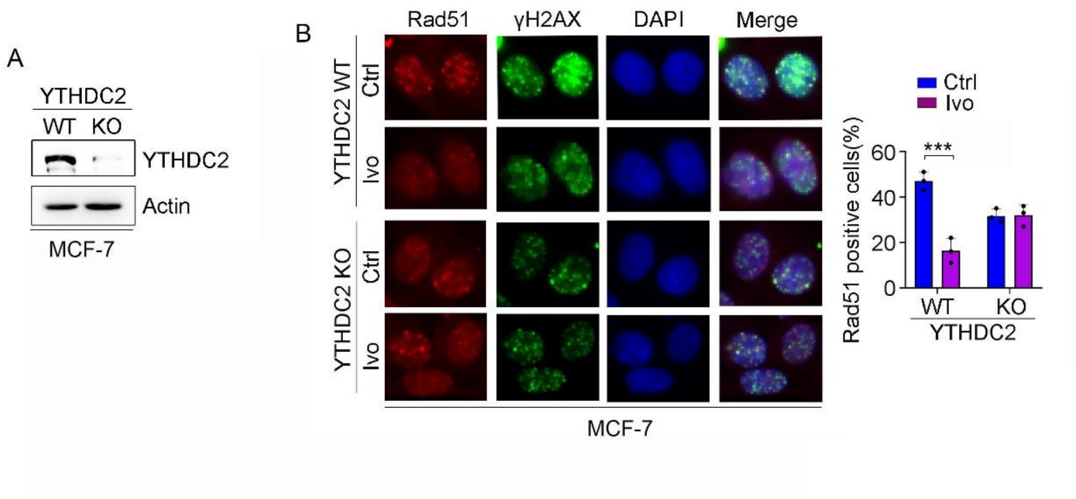

Figure S3. Depletion of YTHDC2 diminishes Ivosidenib-mediated suppression of HR repair. (A) Western blot analysis of YTHDC2 protein levels in MCF-7 wild-type (WT) or knockout (KO) cells. (B) WT or YTHDC2 KO MCF-7 cells were pre-treated with or without 10 μM Ivosidenib(Ivo) for 2 hours, then cells were irradiated with 2 Gy X-ray, and Rad51/rH2AX foci formation were then analyzed. Data were presented as means ± SD. ***p<0.001 by 2-tailed t-test.
